# Salvage robot-assisted radical prostatectomy following focal ablation with irreversible electroporation: feasibility, oncological and functional outcomes

**DOI:** 10.1186/s12894-022-00978-w

**Published:** 2022-03-02

**Authors:** Alexandar Blazevski, William Gondoputro, Matthijs J. Scheltema, Amer Amin, Bart Geboers, Daniela Barreto, Anne-Maree Haynes, Ron Shnier, Warick Delprado, Shikha Agrawal, James E. Thompson, Phillip D. Stricker

**Affiliations:** 1 St. Vincent’s Prostate Cancer Research Centre, Darlinghurst, NSW Australia; 2 Garvan Institute of Medical Research and Kinghorn Cancer Centre, Darlinghurst, NSW Australia; 3 St. Vincent’s Clinical School, University of New South Wales, Sydney, Australia; 4 Department of Urology, Amsterdam UMC, Amsterdam, The Netherlands; 5 Department of Interventional Radiology, Amsterdam UMC, Amsterdam, The Netherlands; 6 I-MED Radiology, Sydney, NSW Australia; 7 Douglas Hanly Moir Pathology, Macquarie Park, NSW Australia

**Keywords:** Focal therapy, Salvage prostatectomy, Irreversible electroporation, IRE, Prostate cancer

## Abstract

**Background:**

To report the feasibility, oncological and functional outcomes of salvage robot-assisted radical prostatectomy (sRARP) for recurrent prostate cancer (PCa) after irreversible electroporation (IRE).

**Methods:**

This was a retrospective analysis of patients who underwent sRARP by a single high-volume surgeon after IRE treatment in our institution. Surgical complications, oncological and functional outcomes were assessed.

**Results:**

15 patients with at least 12 months follow up were identified out of the 234 men who underwent primary IRE between 2013 and 2019. The median [IQR] age was 68 (62–70) years. The median [IQR] time from focal IRE to sRARP was 42 (21–57) months. There were no rectal, bladder or ureteric injuries. The T-stage was pT2 in 9 (60%) patients and pT3a in 6 (40%) patients. Only one (7%) patient had a positive surgical margin. At a median [IQR] follow up of 22 (16–32) months no patient had a biochemical recurrence (PSA > 0.2). All 15 patients were continent (pad-free) by 6 months and 9 (60%) patients had erections sufficient for intercourse with or without PDE5 inhibitors. No predisposing factors were identified for predicting erectile dysfunction after sRARP.

**Conclusions:**

In patients with recurrent or residual significant PCa after focal IRE ablation it is feasible to obtain good functional and oncological outcomes with sRARP. Our results demonstrate that good outcomes can be achieved with sRARP, when respecting close monitoring post-IRE, good patient selection and surgical experience. The limitations of this study are that it is a small series, with short follow up and a lack of standardised quality of life instruments.

**Supplementary Information:**

The online version contains supplementary material available at 10.1186/s12894-022-00978-w.

## Introduction

Focal ablation (FA) is an emerging treatment option for select men with localised prostate cancer (PCa) [1, 2]. A number of different FA energy modalities are currently available, each with varying characteristics [3]. They have been shown to considerably reduce urinary, sexual and bowel side effects when compared to conventional whole-gland treatments, while still having acceptable short to medium term oncological control [4–9]. A significant proportion do, however, experience local recurrence. The ability to achieve comparable outcomes with salvage radical treatment to primary radical treatment is therefore an essential pre-requisite before focal ablation is widely embraced by urologists and endorsed by ‘best practise’ guidelines.

Irreversible electroporation (IRE) ablates prostate tissue by delivering a high-voltage electric current between transperineally inserted electrodes [10]. Recent literature has demonstrated that IRE provides good short to medium-term oncological control with minimal impact of quality of life (QoL) [7, 8]. However, despite the satisfactory outcomes, there are still a significant number of patients that recur locally and need salvage treatment (Table 1) with re-do focal ablation, robot-assisted radical prostatectomy (sRARP) or radiotherapy.

**Table 1:** Salvage treatments for patients treated with IRE ablation for the primary treatment of prostate cancer

234 Patients underwent IRE ablation as primary treatment for prostate cancer	
Re-do IRE ablation	n = 27
Radiotherapy	
EBRT	n = 4
Brachytherapy	n = 2
Radical Prostatectomy	n = 19
Our institution	n = 17
Our Institution with 12 months follow-up post-surgery	n = 15
Other institution	n = 2

Currently there is only limited data on sRARP in men who have failed FA treatments [11–15]. While recent literature suggests that sRARP is feasible and has reasonable outcomes, no study has investigated the outcomes of sRARP in men after FA with IRE. The aim of this present study is to assess the feasibility, oncological and functional outcomes of patients treated with focal IRE who subsequently underwent sRARP at our centre.

## Patients and methods

### Study design

Following institutional review board approval (HREC approval SVH 13/018 and 16/110), data were retrieved from a single centre (St. Vincent’s Prostate Cancer Centre, Sydney, Australia) prospective database of patients treated with primary focal IRE between February 2013 and January 2020.

Patients that underwent a sRARP at our institution after at least one prior IRE procedure were included in the analysis. Patients were only included in the study if they had at least 12-month follow-up from the sRARP. No patient underwent an open radical prostatectomy in this series. We included consecutive patients with histopathological confirmed residual or recurrent clinically significant PCa. Salvage treatment (sRARP, radiotherapy or re-do IRE ablation) was offered to patients whose post-IRE biopsy showed clinically significant PCa (Gleason grade group ≥ 2) (Table 1). It was an institution policy to offer a maximum of two focal therapy sessions per patient. All patients are offered a consultation with a radiation oncologist independently.

Indications for sRARP in this setting included -

1. Multifocal intermediate risk prostate cancer following IRE
2. Patients’ decision not to undergo further focal ablation therapy
3. Two treatments of focal ablation with further significant recurrence
4. Significant upgrading in PCa not amenable to further focal ablation
5. Physicians lack of comfort with persisting with focal therapy.

Metastatic disease was excluded by prostate-specific membrane antigen (PSMA)/positron emission tomography (PET) scan. All patients had a life expectancy greater than 10 years. Patients who previously received androgen deprivation therapy or radiotherapy were excluded. All sRARP’s were performed by a single surgeon (PS). Decisions about neurovascular bundle preservation were made by the surgeon based on intraoperative findings, prior imaging and biopsy. A pelvic lymph node dissection was performed, where appropriate according to Briganti nomogram (> 5 risk of LNMs). All pathological specimens were reviewed by a dedicated uro-pathologist (WDP).

In our institution, patients undergoing FA with IRE are monitored rigorously during follow up with serial PSA levels (every three months for the first year and then every six months thereafter). All patients undergo a T1/T2 MRI within a week of treatment to ensure adequate ablation of the intended area and then a multiparametric MRI (mpMRI) at six months post treatment to assess for recurrence/residual disease. Patients subsequently undergo a transperineal biopsy (template with targeted biopsies of ablated area) at 12 months (or earlier if suspicion on MRI) to assess for oncological control. Follow up schedule or salvage treatment is dictated thereafter by the 12-month post treatment biopsy.

### Data collection

IRE data was collected prospectively for each patient. The data that was collected included: age at IRE ablation, PSA level prior to treatment, pre-treatment biopsy Gleason score, mpMRI, area ablated, ablation treatment parameters including number of electrodes, volts and sets, post treatment PSA levels, mpMRI and biopsy results. It was also recorded if a patient had a second IRE ablation prior to prostatectomy.

For each patient data for sRARP was collected retrospectively. This data included: age at surgery, date of surgery, pre-treatment PSA level and biopsy Gleason score, histopathological data from sRARP, operative data, post-operative complications, follow-up PSA levels, need for adjuvant/salvage treatment and patient-reported continence and erectile function measures (collected by independent data managers).

Urinary continence was strictly defined as self-reported lack of need for continence pads (0 pads usage). Patients were considered potent when they had self-reported erections firm enough for penetrations with or without the use of a phosphodiesterase type 5 inhibitor.

### Statistical analysis

Quantitative variables are shown in median and interquartile (IQR) scores. A Mann–Whitney U test was used to compare pre-operative parameters with the development of erectile dysfunction after sRARP. Statistical analysis was done with the R statistical environment, versions 3.1.0. Statistical significance was considered *p* < 0.05.

## Results

A total of 15 patients were included in the analysis. The median (IQR) age of the cohort was 68 (62.5–70) years. The median (IQR) time from focal IRE ablation to sRARP was 42 (21–57) months. The median (IQR) PSA level prior to surgery was 6.6 (4.1–8.4) ng/mL (Table 2).

**Table 2:** Preoperative characteristics

Number of patients	15
Age at sRARP, years, median (IQR)	68 (62–70)
PSA level before focal IRE, ng/ml, median (IQR)	4.9 (5.2–6.7)
Gleason Grade/ISUP Group before FT, n (%)	
3 + 3/1	1
3 + 4/2	13
4 + 3/3	1
Number of ablations	1
Number of electrodes	4 (4–6)
Number of sets	(6–10)
Maximum voltage	2500 (2250–2660)
Minimum voltage	1350 (1200–1500)
IRE Ablation site, n %	
Posterior Base to Mid	7
Posterior Apex to Mid	4
Posterior Base to Apex	1
Anterior Base to Mid	2
Anterior Base to Apex	1
Number of re-do ablations for recurrence	4
PSA level before sRARP, ng/ml, median (IQR)	6.6 (4.1–8.4)
Location of recurrence, n (%)	
Infield	2
Adjacent	2
Out-of field	11
Erectile function sufficient for intercourse	14/15 (93.3%)

sRARP was performed in all patients without any conversion to open surgery during the procedure. Bilateral nerve sparing was performed in 9 patients (60%) and unilateral or partial nerve sparing in 6 patients (40%). There were no rectal, bladder or ureteric injuries in this series. Pelvic lymph node dissection (PLND) was only performed in 4 patients (27%). Dissection difficulty attributed to prior focal ablation with IRE was mentioned in 12 patients (80%). The median prostate volume (IQR) was 56 (43–70) mL. The median bloods loss was 200 mL and median length of stay was two days (Table 3).

**Table 3:** Operative and follow-up details

Prostate vol, median (mL) (IQR)	56 (43–70)
Index tumour vol, median (mL) (IQR)	0.75 (0.25–1)
Time between initial IRE ablation and sRARP (mo), median (IQR)	42 (21–57)
Nerve sparing, n (%)	
Unilateral	6 (40%)
Bilateral	9 (60%)
None	0
Pelvic lymph node dissection, n (%)	4 (27%)
Gleason Grade/ISUP GGG after sRP, n (%) [Index Lesion]	
3 + 3/1	0
3 + 4/2	8 (53%)
4 + 3/3	1 (7%)
4 + 4/4	3 (20%)
4 + 5/5	3 (20%)
Gleason Grade/ISUP GGG after sRP, n (%) [Composite]	
3 + 3/1	0
3 + 4/2	9 (60%)
4 + 3/3	3 (20%)
4 + 4/4	0
4 + 5/5	3 (20%)
Surgical margin involvement, n (%)	1 (7%)
T stage, n (%)	
pT2	9 (60%)
pT3a	6 (40%)
pT3b	0
Presence of cribriform pattern, n (%)	
Yes	5 (33%)
No	9 (67%)
Surgical complications, n (%) (rectal injury, cystotomy, ureteric injury)	0
Blood loss, mL, media (IQR)	200 (200–250)
Length of Stay, median	2 days
Follow-up (mo) since sRARP, median (IQR)	20 (15–30)
Postoperative RT, n (%)	0
Metastasis, n (%)	0
Continence, n (%)	
Pad free after 3 months	14
Pad free after 6 months	1
Incontinent	0
Erections, n (%)	
Potent	5 (33%)
Potent with PDE-5 inhibitor	4 (27%)
Impotent	6 (40%)

In terms of oncological outcomes, the Gleason grade was 3 + 4 = 7 (grade group 2) in eight patients (53%); 4 + 3 = 7 (grade group 3) in one patient (7%); 4 + 4 = 8 (grade group 4) in three patients (20%) and 4 + 5 = 9 (grade group 5) in three patients (20%). The T-stage distribution was pT2 in nine patients (60%) and pT3a in six patients (40%). Of the four patients that had a PLND, only one had a single positive lymph node. An in-field recurrence was found in two patients (13%), adjacent PCa to the ablation zone was found in two patients (13%) and an out-of-field recurrence in 11 patients (73%). A positive surgical margin (PSM) was present in one patient (7%). The length of the PSM in this patient was 1.5 mm and the Gleason grade was 3. When compared to biopsy prior to sRARP, one patient (7%) had Gleason grade group upgraded while two patients (14%) had it downgraded.

### Patient characteristics before IRE

The median PSA (IQR) prior to initial focal IRE ablation was 4.9 (5.2–6.7) ng/mL. The Gleason grade was 3 + 3 = 6 (grade group 1) in one patient (7%), 3 + 4 = 7 (grade group 2) in 13 patients (87%) and 4 + 3 = 7 (grade group 3) in one patient (7%). All patients had one IRE ablation in the initial treatment. Four patients (27%) had a re-do IRE ablation as a separate procedure. The median (IQR) number of electrodes uses was 4 (4–6) and the number of sets (IQR) was 6 (6–10). The median (IQR) maximum voltage applied during IRE ablation was 2500 (2250–2660) and the minimum voltage was 1350 (1200–1500). Anterior ablation was performed in three patients (20%) while posterior ablation in 12 patients (80%). The apex of the prostate was included in 6 patients (40%).

At a median (IQR) follow-up of 22 (16–32) months, no patient developed biochemical recurrence. No patient has developed metastatic disease or died. No patient received post-operative radiotherapy (Table 4).

**Table 4:** Follow up details

Follow-up (mo) since sRARP, median (IQR)	22 (16–32)
Postoperative RT, n (%)	0
Metastasis, n (%)	0
Continence, n (%)	
Pad free after 3 months	14
Pad free after 6 months	1
Incontinent	0
Erections, n (%)	
Potent	5 (33%)
Potent with PDE-5 inhibitor	4 (27%)
Impotent	6 (40%)

In terms of functional outcomes, 14 men (93%) were pad-free by 3 months and all men were pad-free by 6 months. Erectile function sufficient for intercourse with or without a PDE-5 inhibitor was preserved in 9 (60%) patients (Table 5).

**Table 5:** Mann–Whitney U test showing no predictive factors for erectile dysfunction after sRARP

	Erection sufficient for intercourse (n = 9)	Erections insufficient for intercourse (n = 6)	*P*
Age (median, IQR)	62 (60–68)	65 (62–67)	0.712
Baseline EPIC sexual score (median, IQR)	77.325 (68.73–84.13)	68.27 (60.59–76.925)	0.894
Redo Ablation	1 (11%)	3 (50%)	0.690
Prior PDE-5 use before IRE	0 (0%)	2 (33%)	0.299

### Procedure description

All men underwent sRARP using the Da Vinci platform. The sRARP procedure was performed as a routine anterior, transperitoneal RARP except that the area previously ablated with IRE was dissected last. Fibrosis is always encountered in the ablated area. It is dissected last to help align the anatomy as accurately as possible and increase mobility of the prostate to allow an improved nerve spare on that side of the prostate. The ablated area is difficult to dissect and requires slow, careful dissection. While every attempt is made to preserve the neurovascular bundle, the dissection in the ablated area is wider and may inevitably include the nerves. Prior imaging and biopsy also aides in determining how wide to make the dissection on the treated side. During the procedure every attempt is made to preserve the bladder neck and maximise urethral length. A bladder neck plication stitch is utilised in the aim to improve recovery of urinary continence.

## Discussion

The long natural history of intermediate risk PCa and the extremely low 10-year mortality rate, even with observation (Protect, PIVOT), has made focal ablative treatments an attractive option to reduce the risks and QoL effects of treatment in this group [16–18]. However, whilst this treatment modality has been embraced by a small number of urologists globally there is still considerable concern regarding the possibility of worse outcomes after subsequent radical whole-gland treatments if focal treatment fails. Currently there is limited data regarding sRARP in patients post focal therapy, with the majority of studies reporting oncologic and functional outcomes that appear inferior to those of primary nerve-sparing RARP, LDR brachytherapy and EBRT/ IMRT for favourable intermediate-risk disease [11–15, 19, 20].

These studies have reported on patients previously treated with high-intensity focused ultrasound (HIFU), cryotherapy or vascular-targeted photodynamic therapy. This present study reports the first series of sRARP in patients undergoing prior focal IRE ablation.

For instance, Thompson et al. showed that sRARP post HIFU ablation was safe but had sub-optimal continence, sexual and oncological outcomes when compared to primary RARP [19, 20]. In this series only 65.5% of men were pad-free by 12 months post sRARP. Furthermore T3a/b disease was found in 64.5% of patients and positive surgical margin in 44.4%. These results may be explained by different surgeons performing the focal HIFU ablation to the surgeons performing the sRARP procedure. The results in our current series may be superior because the patients received more intense follow up post focal ablation which included 3-monthly PSA, 6-month mpMRI and 12-month transperineal biopsy.

Additionally, another series investigating RP post focal ablation (with most men receiving HIFU or laser ablation) showed that T3 disease was found in 59% of men and 38% had a positive surgical margin. In this study, 91.2% of men were pad-free at last follow up. Importantly, there was only a median interval of 10 months between focal ablation to salvage radical prostatectomy in this series suggesting that the men may not have been suitable for focal ablation in the first instance. Also, the majority (82.4%) of these men had an open radical prostatectomy [11].

In another study, Pierrard et al., showed that sRARP post vascular-targeted photodynamic therapy was safe and feasible. Here 31% of men had T3 disease and 31% had positive surgical margins. Furthermore 75% of men recovered potency and 88% of men required 1 pad or less at 12 months post treatment. The superior functional outcomes in this study may be secondary to the focal ablation being of a non-thermal modality [13].

Furthermore, a recent prospective pair matched study by Bhat et al. compared sRARP post focal ablation compared to primary RARP patients. Here the authors showed that the primary RARP patients were more likely to have a nerve sparing procedure and that the sRARP group had a higher incidence of positive surgical margin (40% vs. 15%) [21].

Primary radical prostatectomy (RP), either robotic or open, is a common procedure with known but acceptable complications including incontinence and erectile dysfunction [22]. However, it is well recognised that salvage RP after radiotherapy or focal ablation is a difficult operation that can have significant morbidity and requires considerable experience and technical ability to perform [23, 24].

It is currently unknown how to definitively monitor patients who have had prior FA regardless of the modality used. There are varying follow-up schedules used around the world. In our institution we monitor patients with 3-monthly PSA levels for the first year, a mpMRI at 6 months and then a transperineal template biopsy with targeted biopsies of the ablated area at 12 months post treatment. If biopsy is negative, then patients are monitored with 6-monthly PSAs, imaging at 12–24 months and repeat biopsy in 2–4 years. A positive biopsy is defined as prostate cancer ≥ Gleason 3 + 4. Patients with recurrent or residual PCa have a discussion about re-do IRE ablation (maximum of 2 ablations) or definitive whole-gland therapy.

In this current study 15 men underwent sRARP for recurrent disease after focal IRE ablation. This study has demonstrated that sRARP is safe and feasible in men experiencing recurrent disease post focal ablation with IRE. The positive margin rate of 7% and extracapsular disease of 40% show that the oncological results are comparable to those of primary RP.

Furthermore, these results are superior to other recent studies investigating salvage RP in the post focal ablation setting. There are a number of reasons why this may be the case. Firstly, the intensive follow-up of the FA patients allows for early identification of recurrence or residual disease. While mpMRI is useful in the monitoring of FT patients, it has a low NPV and therefore, we believe that a biopsy is essential in the surveillance of these patients [25].

Focal therapy centres that only use PSA and MRI-based follow up without routine biopsy may compromise the opportunity for cure in a small subset of patients unless a follow-up biopsy is performed, due to the lower sensitivity of mpMRI post-FA as a result of extensive treatment artefact which severely distorts the T2 and DWI sequences that are the foundation of mpMRI prostate analysis. With the development of novel prostate imaging such as PSMA PET/CT, the NPV of combined MRI + PSMA imaging-based surveillance protocols may be higher in the future and reach a point where routine biopsy is no longer required [26].

Secondly, it is important to note that in this series the same surgeon who performed all IRE ablations also performed all sRARP’s. This differs to many other centres where the urologist who performs FAs are not the same as those who perform sRARP’s. Such separation of treatment teams may lead to ‘reluctance’ to biopsy and refer treatment failures, similar to the well documented under-referrals and delayed referrals by urologists to radiation oncologists for biochemical recurrence after RARP. The unique fact that a single urologist (PS) was able to offer FA and was highly experienced in sRARP at our centre may have led to superior PSM and ECE outcomes in the cohort as the surgeon had a lower threshold to operate. Therefore, we suggest that surgeons who offer FA treatments, have a close relationship with a surgeon that performs sRARP (Additional files 1, 2).

One important finding from this study is that despite a negative follow up biopsy post-treatment, some patients still recur. Therefore, ongoing active surveillance of this cohort is encouraged. Four patients with negative post treatment biopsies and imaging later developed high risk prostate cancer (median time post IRE treatment was 48 months). In these patient’s PSA velocity was the trigger for biopsy.

The importance of early salvage surgery has also been shown in patients with radiation recurrent PCa. Bianco et al. showed that salvage prostatectomy was associated with superior local control if the pre-operative PSA was less than 4 ng/mL [27]. While the PSA nadir post radiation is far lower than in focal ablation, the pre-operative PSA level was still relatively low in our cohort (PSA 6.6 ng/mL).

Functional outcomes were similar to those from our previously reported primary RARP series with all patient’s continent by 6 months and sexual function was preserved in the majority of patients [28]. There were no obvious predictive factors identified for erectile dysfunction after sRARP. However, it is important to point out that the numbers in this cohort are very low and no conclusion can be made from this analysis. It has previously been shown that there is no correlation between prostate segment treatment with IRE and erectile dysfunction or continence [29].

This study had several limitations. Firstly, this is a small, single-centre, single-surgeon retrospective study with short median follow-up of 22 months. Secondly, the surgeon was very experienced in terms of radical prostatectomy overall (> 7000), robotic prostatectomy (> 2500), salvage prostatectomy (> 50) and IRE focal ablation (> 300). Therefore, these results may not be applicable to all centres or urologists. Also, there is bias in this paper in that the patients selected for sRARP were likely chosen given the aggressiveness of their disease. Patients opting for re-do IRE ablation or radiotherapy were not included in this study and therefore does not give an accurate representation of all patients that recur post IRE. Future multi-centre and multi-surgeon studies and the inclusion of patients undergoing other salvage treatments (i.e. repeat IRE and salvage radiotherapy) would give a greater understanding of the management of patients with local recurrence after focal IRE. Lastly the functional outcomes were collected retrospectively from contemporaneous patient records at the time of post-operative consultations with the surgeon and clinical nurse specialist, given that patients at our institution only completed validated questionnaires post IRE ablation and not post radical surgery.

In conclusion, sRARP after focal IRE by an experienced surgeon is feasible, safe and may achieve acceptable oncological and functional outcomes which may be comparable to primary RARP. sRARP should therefore be considered as a suitable treatment option for selected patients with recurrent or residual disease following focal IRE ablation. Intensive follow-up post focal treatment and a low threshold to progress to radical treatment appears essential to achieving good outcomes.

## Supplementary Information

**Additional file 1.** Fibrosis encountered during salvage robot assisted radical prostatectomy in patient that has had previous focal IRE ablation.

**Additional file 2.** Difficult apical prostate dissection during salvage robot assisted radical prostatectomy following previous ablation with focal IRE.

## Data Availability

The data analyses generated and available within the presentenced study, but specific datasets are kept confidential due to their nature of including clinical, pathological and radiological details. Further information regarding dataset analysis may be available from the corresponding author upon request.
